# Inhibition of microtubule dynamics impedes repair of kidney ischemia/reperfusion injury and increases fibrosis

**DOI:** 10.1038/srep27775

**Published:** 2016-06-08

**Authors:** Sang Jun Han, Ji-Hyeon Kim, Jee In Kim, Kwon Moo Park

**Affiliations:** 1 Department of Anatomy, Cardiovascular Research Institute, and BK21 Plus, Kyungpook National University School of Medicine, Daegu 700-422, Republic of Korea; 2 Department of Molecular Medicine and MRC, College of Medicine, Keimyung University, Daegu 704-701, Republic of Korea

## Abstract

The microtubule cytoskeleton is composed of α-tubulin and β-tubulin heterodimers, and it serves to regulate the shape, motility, and division of a cell. Post-translational modifications including acetylation are closely associated with the functional aspects of the microtubule, involving in a number of pathological diseases. However, the role of microtubule acetylation in acute kidney injury (AKI) and progression of AKI to chronic kidney disease have yet to be understood. In this study, ischemia/reperfusion (I/R), a major cause of AKI, resulted in deacetylation of the microtubules with a decrease in α-tubulin acetyltransferase 1 (α-TAT1). Paclitaxel (taxol), an agent that stabilizes microtubules by tubulin acetylation, treatment during the recovery phase following I/R injury inhibited tubular cell proliferation, impaired renal functional recovery, and worsened fibrosis. Taxol induced α-tubulin acetylation and post-I/R cell cycle arrest. Taxol aggregated the microtubule in the cytoplasm, resulting in suppression of microtubule dynamics. Our studies have demonstrated for the first time that I/R induced deacetylation of the microtubules, and that inhibition of microtubule dynamics retarded repair of injured tubular epithelial cells leading to an acceleration of fibrosis. This suggests that microtubule dynamics plays an important role in the processes of repair and fibrosis after AKI.

Microtubules are one of the primary components of the cytoskeleton, and the microtubule network within the cell plays an essential role in the regulation of cell shape and structure, cell division, and cell motility. The microtubule is composed of heterodimers containing α-tubulin and β-tubulin subunits. Diversification of microtubules is the result of post-translational modifications such as polyglycylation, detyrosination, polyglutamylation, and acetylation^1^. These post-translational modifications are closely associated with the functional aspects of the microtubule^2^. Among post-translational modifications, tubulin acetylation is associated with microtubule-stabilization and microtubule dynamics. Abnormal tubulin acetylation has been linked to a number of pathological conditions such as cancer, neurological disorders, and heart disease^1^. In kidney tubular epithelial cells, microtubules play a critical role in the maintenance of cell polarity^3^, and their dynamics influence renal function^4^,^5^,^6^. However, the role of these post-translation microtubule modifications on kidney diseases including acute kidney injury (AKI) and chronic kidney disease (CKD) have yet to be understood.

CKD, characterized by fibrosis and disorders in renal function, is a common clinical problem with increasing incidence and serious clinical consequences^7^. Ischemia/reperfusion (I/R) insult in the kidney is a major cause of AKI, which is a risk factor for CKD. The progression from I/R injury and AKI to subsequent kidney fibrosis is dependent upon sequential changes within the kidney following I/R insult. The initiation phase of I/R induces mild injury, with a loss of brush borders and disorganization of the cytoskeleton leading to tubule cell dysfunction. If the injury is alleviated by appropriate treatment at this stage, tubule cell structure and renal function will be restored. Left untreated, the injury can progress to tubular cell apoptosis and necrosis, tubule cell desquamation into the lumen, luminal congestion and obstruction, and inflammatory responses. Subsequently, AKI enters into a maintenance phase in which the processes of tubule cell death and restoration are occurring simultaneously as a result of proliferation and differentiation of viable tubule cells. If the repair is incomplete, the kidney enters a fibrotic phase characterized by tubule dilatation and atrophy, with expansion of the interstitial space through the accumulation of extracellular matrix, myofibroblasts, and inflammatory cells^8^. These sequential changes in response to AKI are strongly correlated with the disorganization, disruption, and aberrant function of the tubule cell cytoskeleton, including the microtubules^9^,^10^. Several studies have demonstrated that cellular stress is associated with post-translational modifications of microtubules and the microtubule network^11^,^12^. However, the influence of these alterations on the progression from AKI to CKD remains unclear.

Zhang *et al.* demonstrated that stabilization of renal microtubules by tubulin polymerization and cell cycle arrest suppresses the progression of renal fibrosis in a rat unilateral ureteral obstruction (UUO) model^4^, and mitigates lipopolysaccharide-induced AKI by inhibiting Toll-like receptor 4 (TLR4)^13^. Furthermore, Abbate *et al.* reported that I/R injury in the kidney disrupts the microtubule network^14^. These reports suggest that the microtubule network plays an important role in the progression from AKI to CKD. Therefore, we used a mouse I/R injury model and paclitaxel (taxol) treatment to investigate the alteration of microtubule acetylation following an I/R event, and the role of microtubule stabilization in tubular epithelial cell restoration and fibrosis. In this study, we demonstrated that AKI resulting from I/R injury induced microtubule deacetylation, inhibited microtubule dynamics, delayed tubule cell recovery and exacerbated fibrosis. These results suggest that regulation of tubulin acetylation and deacetylation, which can regulate microtubule dynamics, could be considered a therapeutic strategy for AKI and CKD treatment.

## Results

### Ischemia/reperfusion induces deacetylation of α-tubulin in the kidney

**Figure 1:**
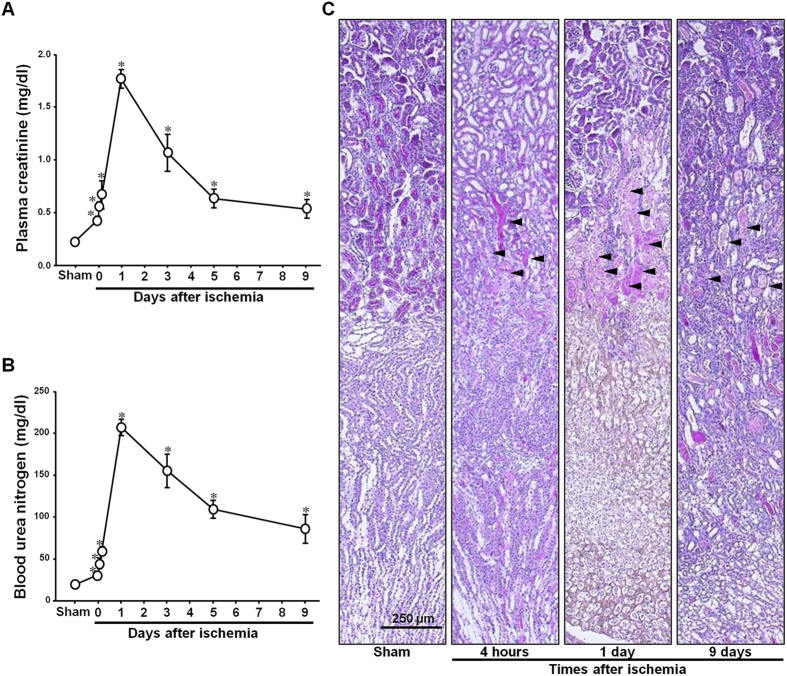
Kidney functional and morphological changes after ischemia/reperfusion injury. Mice were subjected to either 30 min of ischemia or sham operation. (**A**) Plasma creatinine (PCr) and (**B**) blood urea nitrogen (BUN) levels were measured at indicated times. (**C**) Kidney sections were stained with periodic acid-Schiff (PAS) reagent as described in the Materials and Methods. Arrowheads indicate injured tubules. Results are expressed as mean ± SEM (n = 5). *p < 0.05 vs. Sham.

To confirm that the I/R procedure induced AKI in the mice, we determined PCr and BUN concentrations. Thirty minutes of bilateral renal ischemia followed by reperfusion were found to significantly increase PCr and BUN concentrations (Fig. 1A,B). The PCr and BUN concentrations reached a peak 24 h following reperfusion, and then gradually decreased over time (Fig. 1A,B). Consistent with renal functional impairment, I/R induced tubular epithelial cell disruption, congestion, and flattening of tubules, and increased numbers of renal interstitial cells (Fig. 1C).

**Figure 2:**
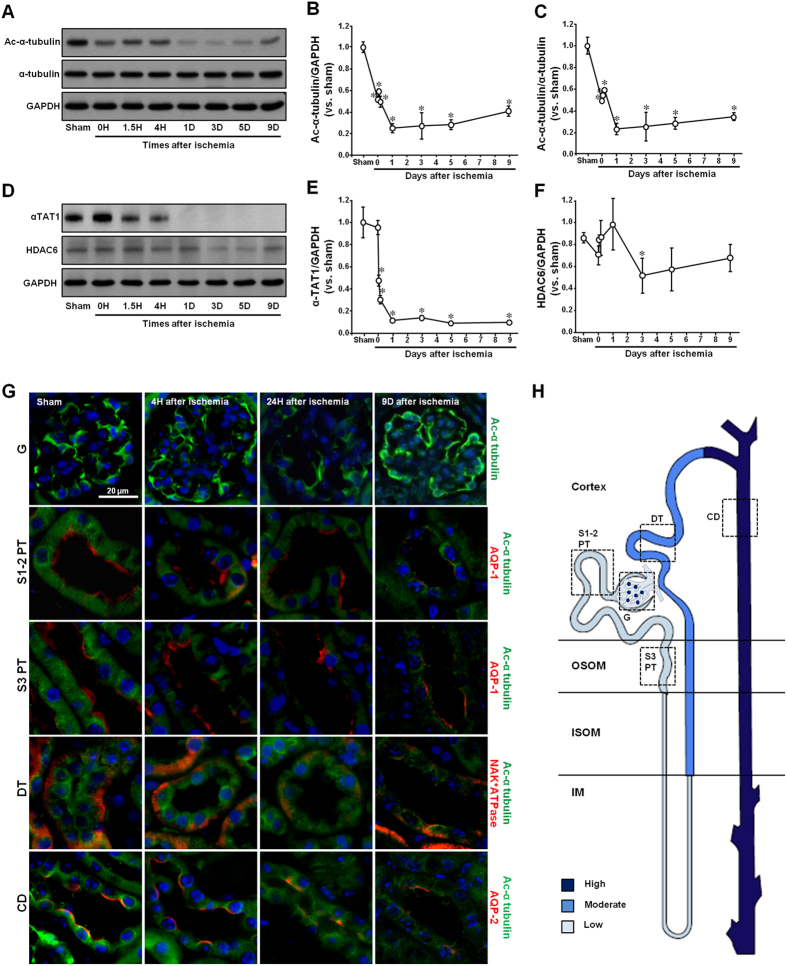
Changes in α-tubulin acetylation and its regulatory protein expression following ischemia/reperfusion injury. Mice were subjected to either 30 min of ischemia or sham operation (Sham). Kidneys were harvested at the indicated times. (**A–F**) Kidney samples were subjected to western blot analysis using anti-acetylated-α-tubulin (**A**), -α tubulin (**A**), -αTAT1 (**D**) and -HDAC6 (**D**) antibodies. GAPDH was used as a loading control. (**B,C,E,F**) Densities of blots were measured using the NIH ImageJ program. (**G**) Kidney sections were subjected to immunofluorescence staining using anti-acetylated-α-tubulin, -AQP1, -AQP2, and -Na/K-ATPase antibodies as described in the Materials and Methods. 4′,6-diamidino-2-phenylindole (DAPI) stain was used to visualize the nucleus. (**H**) Diagram of acetylated α-tubulin expression in normal kidney tubule: (**G**) Glomerulus; S1-2 PT, Segment 1 to 2 in proximal tubule; S3 PT, Segment 3 in proximal tubule; DT, Distal tubule; CD, Collecting duct. Results are expressed as mean ± SEM (n = 3). *p < 0.05 vs. Sham.

In order to determine whether I/R affects microtubule acetylation, we utilized ac-α-tubulin Ab in western blot analysis and immunofluorescence staining to assess serial changes in tubulin acetylation from the initiation to the fibrosis phase of I/R. The concentration of ac-α-tubulin decreased after 30 min of ischemia, recovered slightly between 1.5 and 4 h following reperfusion, decreased again until 5 days post-reperfusion, and finally exhibited partial recovery 9 days after reperfusion (Fig. 2A–C). However, I/R insult did not induce any significant changes in the total levels of α-tubulin expressed (Fig. 2A). This indicates that I/R could regulate α-tubulin acetylation.

Microtubule acetylation and deacetylation are regulated by α-TAT1 and HDAC6, respectively^15^. α-TAT1 applies acetyl residues to lysine 40 (K40) of α-tubulin, whereas HDAC6 removes the acetyl residues from tubulin^16^. αTAT1 expression started to decrease 90 min after reperfusion, and then it almost completely disappeared starting one day after reperfusion, with αTAT1 recovery delayed until nine days post-reperfusion (Fig. 2D,E). In contrast, HDAC6 levels dropped only minimally on the third day following the initiation of I/R, and returned to normal levels after nine days (Fig. 2D,F).

To determine whether localization of acetylated α-tubulin in the kidney tubules is associated with different susceptibility to I/R injury, we determined the distribution of acetylated α-tubulin in the different segments of the renal tubules. In the normal kidney, the proximal tubule, distal tubule, and collecting duct exhibited progressively higher levels of anti-ac-α-tubulin Ab immunoreactivity (Fig. 2G,H). Ac-α-tubulin was highly expressed in the cytoplasm near the nucleus, and oriented in a stripe shape running from the basal to the apical aspect of the tubular epithelial cells (Fig. 2G). The podocytes in the glomerulus exhibited strong immunoreactivity to anti-ac-α-tubulin Ab (Fig. 2G,H). After I/R, the intensity of ac-α-tubulin expression was reduced in the all tubules and renal corpuscles (Fig. 2G). These results indicate that I/R events alter microtubule acetylation, and this process could be associated with the injury and repair mechanisms following I/R in the kidney.

### Following I/R insult, taxol inhibited tubule cell proliferation and accelerated interstitial cell proliferation

**Figure 3:**
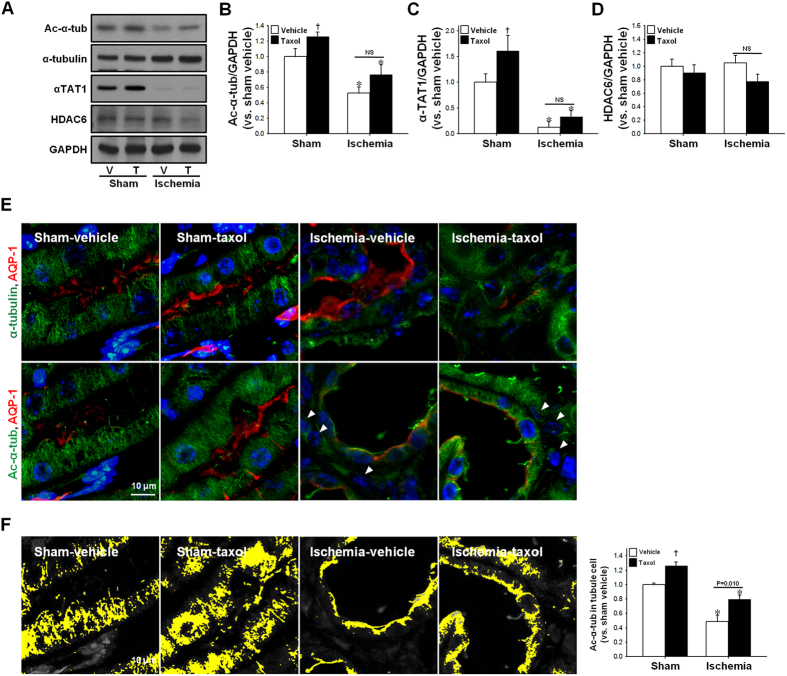
Effect of paclitaxel treatment on microtubule acetylation during the recovery phase following I/R injury. Mice were subjected to either 30 min of bilateral renal ischemia or sham operation (Sham). Mice were treated with paclitaxel (taxol, 300 μg/kg BW) or saline (vehicle) after ischemia or sham beginning on day one following surgery, and continuing every other day until sacrifice. Sixteen days post-procedure, kidneys were harvested and subjected to (**A**) western blot analysis using anti-acetylated α-tubulin (Ac-α-tub), -α-tubulin, -αTAT1, and -HDAC6 antibodies. GAPDH was used as a loading control. V, vehicle; T, taxol. (**B–D**) Band density was measured using the NIH Image J software. (**E**) Kidney sections were subjected to immunofluorescence staining using anti-ac-α-tubulin (green), -α-tubulin (green), and -aquaporin 1 (AQP1, red) antibodies as described in the Materials and Methods. 4′,6-diamidino-2-phenylindole (DAPI, blue) stain was used to visualize the nucleus. Arrow heads indicate ac-α-tubulin-positive interstitial cells. (**F**) Accumulation of ac-α-tubulin-stained microtubules (yellow) in tubule cells was measured using the i-solution software. Results are expressed as mean ± SEM (n = 5). *p < 0.05 vs. respective sham. ^†^*p* < 0.05 vs. vehicle-sham.

Tubulin acetylation accelerates the polymerization of tubulins, stabilizes microtubules, and suppresses microtubule dynamics, thereby leading to inhibition of cell division and differentiation^17^. In order to test the role of microtubule acetylation on the division and differentiation of tubular cells following ischemic injury, taxol treatment (TTx) was administered to mice every other day during the recovery phase after I/R insult. First, we determined whether TTx enhanced microtubule acetylation. TTx in sham mice increased the expressions of ac-α-tubulin without changing the α-tubulin concentration (Fig. 3A,B). TTx increased α-TAT1 levels in the kidney (Fig. 3A,C), but it did not induce a significant change in HDAC6 expression (Fig. 3A,D). Additionally, although not statistically significant, post-I/R reductions in ac-α-tubulin expression were less in the TTx mice (p = 0.078) (Fig. 3A,B). In sham-operated mice, TTx increased the ac-α-tubulin levels as compared to vehicle-treated control mice (p < 0.05) (Fig. 3A,B). Ac-α-tubulin and α-tubulin staining demonstrated that TTx affects the microtubule networks in the renal tubules (Fig. 3E). After I/R injury, microtubules in the kidneys of vehicle-treated control mice lost their vertical orientation, and showed weak signal intensity by virtue of being scattered within the cytoplasm (Fig. 3E). In contrast, microtubules in the TTx mouse kidneys revealed a more intense and regular striped pattern, and the locations of ac-α-tubulin (Fig. 3E, lower lane) and α-tubulin expression were similar (Fig. 3E, upper lane). Aggregation of ac-α-tubulin was greater in the TTx-treated I/R-induced mouse kidney cells than in vehicle-treated I/R-mouse kidney cells (Fig. 3F). These results suggest that tubulin acetylation could inhibit microtubule dynamics.

**Figure 4:**
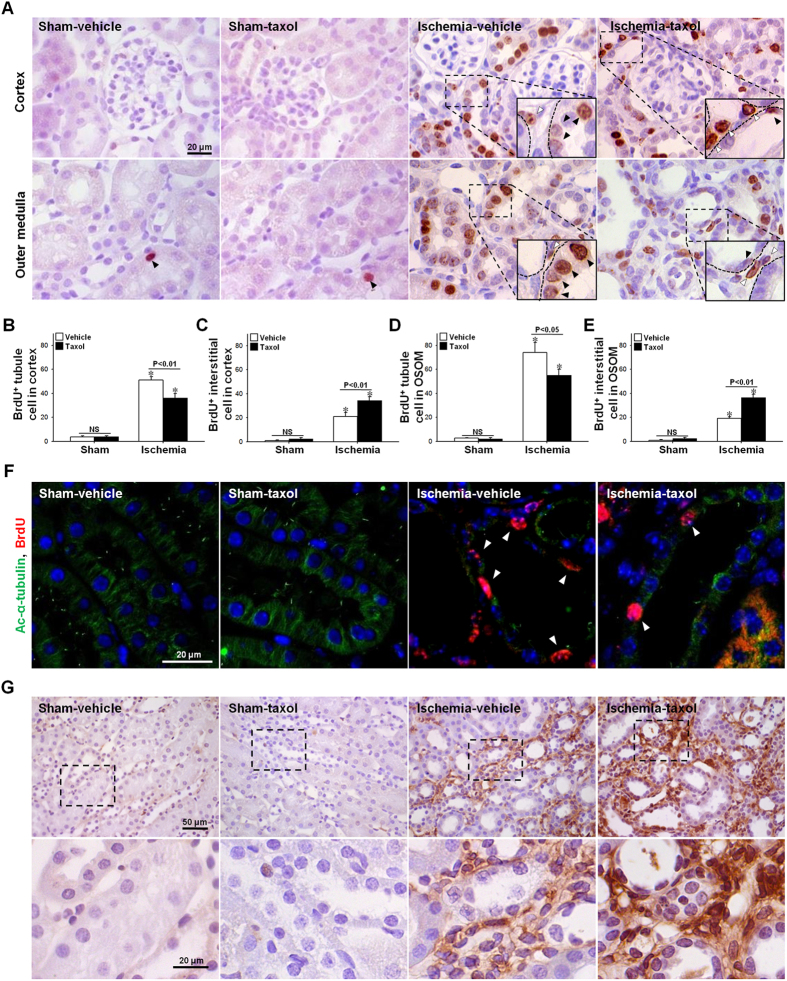
Effect of paclitaxel treatment on the proliferation of tubular epithelial cells and interstitial cells, and cell cycle arrest protein expression during the recovery phase following I/R injury. Mice were subjected to either 30 min of ischemia or sham operation (Sham). Mice were administered paclitaxel (Taxol, 300 μg/kg BW) or saline (Vehicle) beginning on one day following surgery, and continuing every other day until sacrifice. To detect proliferating cells, BrdU was administered to mice beginning on day one following either I/R or Sham, and continuing every other day until sacrifice. Sixteen days post-procedure, kidney sections were immunohistochemically stained using (**A**) anti-BrdU antibody. Hematoxylin stain was used to visualize the nucleus. BrdU-positive (BrdU^+^) tubular cells (**B,D**) and interstitial cells (**C,E**) were counted in the cortical (**B,C**) or outer medullary (**D,E**) regions. Filled and blank arrowheads indicate BrdU-positive tubule and interstitial cells, respectively. (**F**) Kidney sections were subjected to immunofluorescence staining using anti-ac-α-tubulin (green) and anti-BrdU (red) antibodies. 4′,6-diamidino-2-phenylindole (DAPI, blue) stain was used to visualize the nucleus; photomicrographs were obtained from the outer medulla. (**G**) Kidney sections were immunohistochemically stained using anti-PCNA (brown) antibody. Hematoxylin was used to visualize the nucleus (blue), and photomicrographs were obtained from the outer medulla. Lower panels are magnified the inserts. Results are expressed as mean ± SEM (n = 5). *p < 0.05 vs. respective sham.

To define the role of microtubule acetylation on tubule cell proliferation, we determined the total proliferative cell count during the entire recovery phase by means of the BrdU incorporation assay. BrdU was administered daily beginning on day one following the ischemic event. Sixteen days following I/R insult, the number of cells incorporating BrdU in the tubules and the interstitium of kidneys increased significantly as compared to sham (p < 0.01) (Fig. 4A–E). TTx during recovering phase after the peak of injury significantly reduced the post-I/R increase in BrdU-positive tubule cells (p < 0.05) (Fig. 4A,B,D). Interestingly, TTx enhanced the post-I/R increase in the number of BrdU-positive interstitial cells (p < 0.01) (Fig. 4A,C,E). These results indicate that tubule cells and interstitial cells exhibit different responses to TTx.

To determine whether tubulin acetylation is involved in cell proliferation, we double-stained cells with anti-BrdU and anti-ac-α-tubulin Abs. BrdU-positive tubular cells revealed low immunoreactivity to ac-α-tubulin Ab (Fig. 4F), indicating that tubulin acetylation may inhibit cell proliferation. In order to characterize the proliferation of cells at the fibrotic phase, we quantified the PCNA-positive cells in the kidney 16 days after I/R insult. PCNA-positive cells were primarily observed in the interstitium (Fig. 4G), and PCNA-positive cells in TTx treated mice were greater than that in the vehicle-treated mice (Fig. 4G). These results indicate that interstitial cell proliferation is great at late phase, fibrosis phase, after I/R insult, and TTx accelerated the proliferation of these interstitial cells in difference with tubules cells. In previous study we reported that interstitial cell proliferation after I/R insult continuously increased over time^18^.

### Taxol delays functional and histological recovery of I/R-injured kidneys resulting in fibrosis

**Figure 5:**
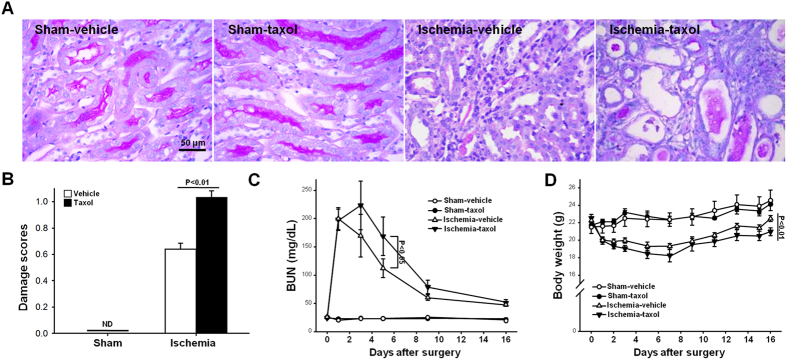
Effect of paclitaxel treatment on functional and morphological kidney recovery after I/R injury. Mice were subjected to either 30 min of ischemia or sham operation (Sham). Mice were administered either paclitaxel (Taxol, 300 μg/kg BW) or saline (Vehicle) from day one following surgery, and continuing every other day until sacrifice. (**A**) Sixteen days post-procedure, kidney sections were stained with periodic acid-Schiff (PAS) reagent as described in the Materials and Methods. (**B**) Tubular damage was scored as described in the Materials and Methods. Blood urea nitrogen (BUN) concentrations (**C**) and body weight (**D**) were measured at the indicated times. Results are expressed as mean ± SEM (n = 5).

Finally, we investigated the effect of tubulin acetylation in the repair and fibrosis processes subsequent to I/R injury. Sixteen days after I/R insult, there was more evidence of tubular epithelial cell disruption, increased congestion and flattening of tubules, and a greater number and area of interstitial cells in the TTx mice as compared to the vehicle-treated control mice (Fig. 5A,B). Consistent with these renal histological results, TTx mice exhibited higher BUN levels than in Control mice. BUN concentration in the vehicle-treated control mice reached a peak one day following ischemic insult, and then gradually decreased, whereas BUN levels in the TTx mice increased continuously until three days post I/R insult, and exceeded the peak levels observed in the vehicle-treated control mice (Fig. 5C). Similar to renal functional recovery, the restoration of body weight following I/R insult was delayed in the TTx mice (Fig. 5D). In the sham mice, TTx did not result in any significant histological or functional changes (Fig. 5A–D).

**Figure 6:**
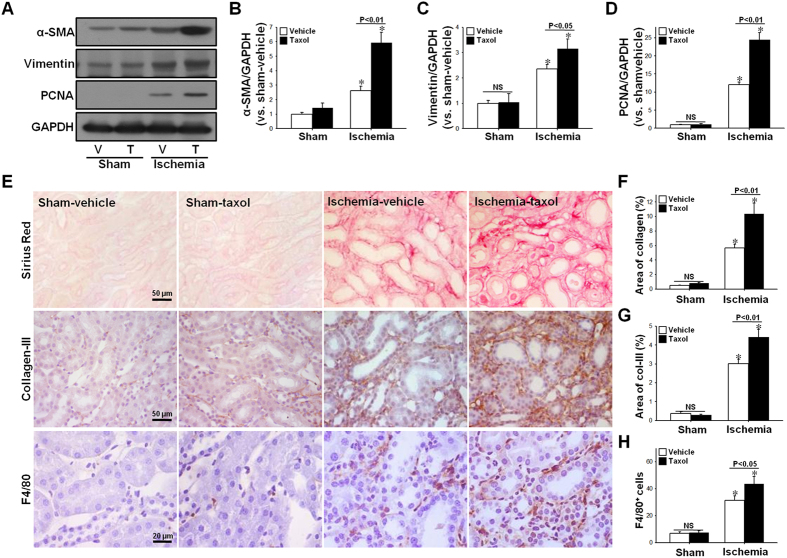
Effect of paclitaxel treatment during recovery phase after I/R injury on progression of fibrosis and macrophage accumulation. Mice were subjected to either 30 min of ischemia or sham operation (Sham). Mice were administered either paclitaxel (Taxol, 300 μg/kg BW) or saline (Vehicle) from day one following surgery, and continuing every other day until sacrifice. (**A**) Sixteen days post-procedure, kidney samples were subjected to western blot analysis anti-α-SMA, -vimentin, -PCNA and -GAPDH antibodies. V, vehicle; T, taxol. (**B–D**) Band density was measured using the NIH ImageJ software. (**E**) Kidney sections were subjected to picro-sirius red (red) staining and immunohistochemical staining using anti-collagen-III (Col-III, brown) or -F4/80 (brown) antibodies. Hematoxylin was used to visualize the nucleus (blue), and photomicrographs were obtained from the outer medulla. Percent area of collagen expression (**F**), percent area of collagen-III expression (**G**), and F4/80-positive (F4/80^+^) cells (**H**) were counted (10 fields per kidney; n = 5). Results are expressed as mean ± SEM (n = 5). *p < 0.05 vs. respective sham.

Post-I/R, α-SMA, vimentin, a marker of cell dedifferentiation, and PCNA, a marker of cell proliferation, were all greater in the TTx mice than in the vehicle-treated control mice (Fig. 6A–D). Collagen deposition and collagen III expression were also greater in the kidneys of the TTx mice than in the vehicle-treated control mice (Fig. 6E–G). These results suggest that TTx delays recovery and accelerates fibrosis. Furthermore, the accumulation of F4/80-positive renal cells in TTx mice was higher than that observed in the vehicle-treated control mice (Fig. 6E,H). This suggests that the delayed recovery of tubule cells may accelerate infiltration of inflammatory cells. Taken as a whole, these data indicate that inhibition of microtubule dynamics by microtubule acetylation delays functional and histological recovery of the kidney following I/R insult, and accelerates fibrosis. This suggests that microtubule acetylation plays a critical role in the fate of the kidney after I/R injury.

### Taxol accelerates post-I/R increase in p-chk2 and p21 expression

**Figure 7:**
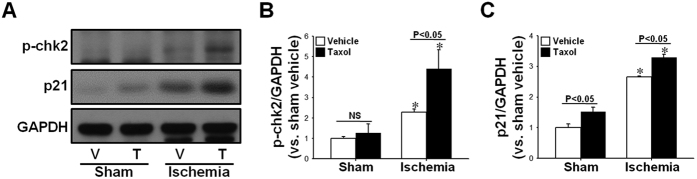
Effect of paclitaxel on I/R-induced cell cycle arrest proteins. Mice were subjected to either 30 min of ischemia or sham operation (Sham). Mice were administered either paclitaxel (Taxol, 300 μg/kg BW) or saline (Vehicle) from day one following surgery, and continuing every other day until sacrifice. (**A**) Sixteen days post-procedure, kidney samples were subjected to western blot analysis using anti-phosphorylated chk2 (anti-p-chk2) and anti-p21 antibodies. GAPDH was used as a loading control. (**B,C**) Band density was measured using the NIH ImageJ software. Results are expressed as mean ± SEM (n = 5). *p < 0.05 vs. respective sham. V, vehicle; T, taxol.

Tubulin acetylation and polymerization has been shown to result in microtubule stabilization and arrest of the cell cycle^17^. Taxol halts the cell cycle at the G0/G1 and G2/M phases and inhibits cell division^19^. Following I/R injury in this experiment, we characterized the expression of the p21 and p-chk2 proteins involved in the induction of G2/M cell cycle arrest. Sixteen days after I/R insult, p21 and p-chk2 expression significantly increased in both vehicle-treated control and TTx mice (p < 0.05) (Fig. 7). These increases in post-I/R p-chk2 and p21 expression in TTx mice were greater than those observed in the vehicle-treated control mice (Fig. 7). These results indicate that microtubule stabilization by TTx induces cell cycle arrest and subsequent inhibition of proliferation in tubular epithelial cells.

### Taxol regulation of microtubule acetylation is cell-type dependent in cultured proximal tubule cells and monocytes/macrophages

**Figure 8:**
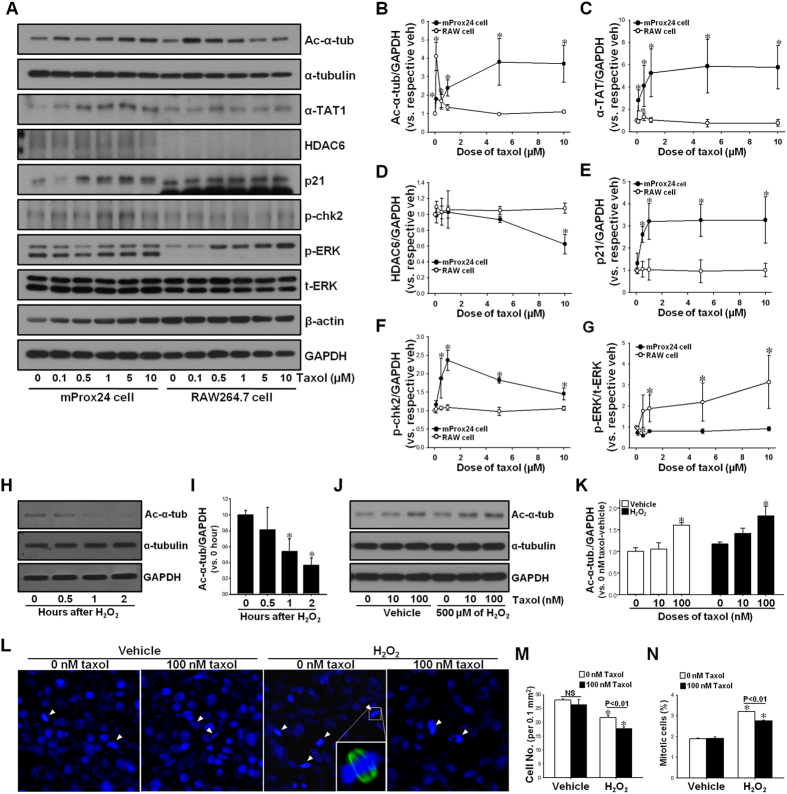
Effect of paclitaxel on tubulin acetylation and cell cycle protein expression on mProx24 and RAW 264.7 cells. Murine proximal tubular epithelial cells (mProx24) and murine macrophage cells (RAW 264.7) cells were treated with 0, 0.1, 0.5, 1, 5, and 10 μM doses of paclitaxel (taxol, T). (**A**) Four hours after treatment, cells were harvested and subjected to Western blot analysis using anti-ac-α-tubulin, -α-tubulin (ac-α-tub), -α-TAT1, -HDAC6, -p21, and -p-chk2 antibodies. β-actin and GAPDH were used as a loading control. (**B–F**) Band density was measured using the NIH ImageJ software. *p < 0.05 vs. respective 0 μM of taxol. (**H**) mProx24 cells were treated with 500 μM of H_2_O_2_ for indicated times. Cell lysates were subjected to Western blot analysis using anti-ac-α-tubulin (ac-α-tub), -α-tubulin, and -GAPDH antibodies. (**I**) Band density was measured using the NIH ImageJ software. *p < 0.05 vs. 0 hour of H_2_O_2_. (**J–N**) Two hours after treatment of 500 μM of H_2_O_2_, cells were incubated in the new medium containing 10 (**J,K**) or 100 (**J–N)** nM of taxol for 18 hours and then applied to Western blot analysis **(J,K**) or immunostaining (**L–N**). (**J,K**) Cell lysates were subjected to Western blot analysis using anti-ac-α-tubulin, -α-tubulin, and -GAPDH antibodies. (**K**) Band density was measured using the NIH ImageJ software. *p < 0.05 vs. respective 0 nM of taxol. (**L**) Fixed cells were subjected to immunofluorescense-staining using anti-α-tubulin (green) antibody. DAPI stain was used to visualize the nucleus (blue). (**M, N**) Total cell number (**M**) and percent of mitotic cells (**N**) were counted as described in Materials & Methods. Results are expressed as mean ± SEM (n = 3). *p < 0.05 vs. respective 0 nM or 100 nM of taxol.

Interestingly, TTx during the recovery phase inhibited post-I/R enhancement of tubule cell proliferation, whereas it accelerated interstitial cell proliferation (Fig. 4). To assess whether this result was cell-type specific, we characterized the response of mProx24 and RAW264.7 cells to TTx. Because most interstitial cells in the fibrotic kidney following I/R injury originate from the bone marrow^20^, we utilized RAW264.7 cells as a representative interstitial cell population. We first determined whether TTx affects acetylation of the microtubule. TTx was found to significantly induce dose-dependent α-tubulin acetylation in the mProx24 cells, whereas α-tubulin acetylation was observed in the Raw264.7 cells with only low doses of taxol (Fig. 8A,B). When we evaluated α-TAT1 and HDAC6 expression, TTx increased α-TAT1 expression in a dose dependent manner in the mProx24 cell, but not in the Raw264.7 cells (Fig. 8A,C). In contrast, TTx decreased HDAC6 expression in the mProx24 cells, but not in the RAW264.7 cells (Fig. 8A,D). With regard to p21 and p-chk2 proteins, TTx increased those expressions in the mProx24 cells in a dose-dependent manner, but not in the Raw264.7 cells (Fig. 8A,E,F). These data indicate that tubular cells have a much greater taxol-mediated anti-proliferative response as compared to inflammatory cells. This suggests that the differences observed in the post-I/R proliferation of murine tubule cells and interstitial cells in the presence of taxol may be associated with cell-type specific responses. Furthermore, we determined whether the regulation of tubulin acetylation affects the regeneration of injured mProx24 cells (mouse proximal tubular cell line cells) after injury. The mProx24 cells were exposed to oxidative stress by H_2_O_2_. Oxidative stress is one of major contributor in I/R injury^21^. Acetylated α-tubulin expression decreased until 2 hours after 500 μM of H_2_O_2_ treatment in a time-dependent manner (Fig. 8H,I). Eighteen-hours after H_2_O_2_ removal the levels of ac-α-tubulin expression returned to normal (Fig. 8J,K). Taxol treatment in both vehicle- and H_2_O_2_-treated cells increased ac-α-tubulin expression (Fig. 8J,K). These data indicate that reduced ac-α-tubulin expression after injury returns to the levels of ac-α-tubulin expression in non-H_2_O_2_-treated cells. In addition, taxol increases acetylation of α-tubulin in both injured and non-injured cells. Eighteen-hours after H_2_O_2_, cell numbers decreased (Fig. 8L,M). This decrease of cell number was greater in taxol-treated cells than in the non-taxol-treated cells (Fig. 8L,M). When mitotic cells were evaluated (Fig. 8L, arrow, note dividing cell in insert, green), mitotic cell number 18 hours after H_2_O_2_ removal was less in the taxol-treated cells than in the non-taxol-treated cells (Fig. 8L,N), indicating that taxol suppresses cell proliferation after H_2_O_2_ insult. Taken together these results suggest that the regulation of tubulin acetylation may regulate recovery of damaged cells.

### Colchicine, an inhibitor of tubulin acetylation, delays tubule cell regeneration and exacerbates kidney fibrosis after I/R

**Figure 9:**
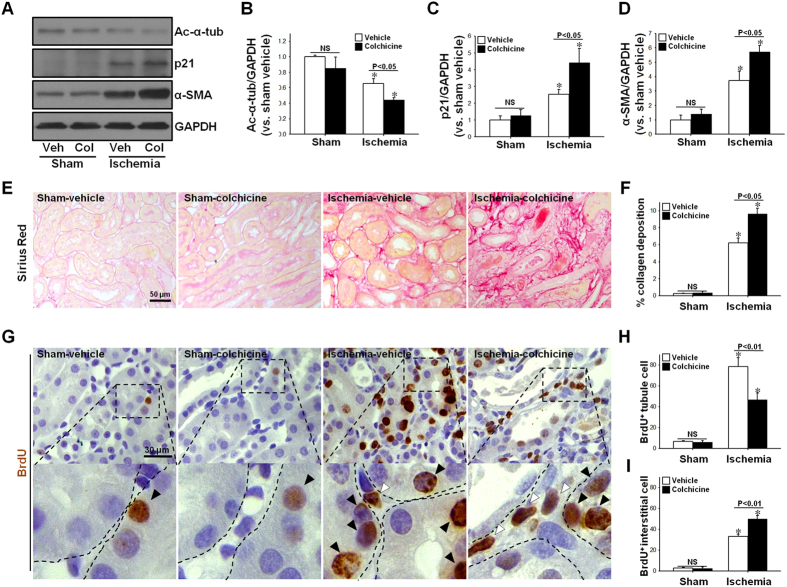
Effect of colchicine treatment during the recovery phase on tubulin acetylation, cell proliferation, and cell cycle arrest, and progression of fibrosis following I/R injury. Mice were subjected to either 30 min of ischemia or sham operation (Sham). Mice were administered colchicine (Col, 100 μg/kg BW) or 0.9% saline (Vehicle, Veh) beginning on one day following surgery, and continuing every other day until sacrifice. (**A**) Kidney samples were subjected to Western blot analysis using anti-ac-α-tubulin (ac-α-tub), -p21, -α-SMA or -GAPDH antibodies. (**B–D**) Band density was measured using the NIH Image J software. (**E–G**) Kidney sections were subjected to either picro-sirius red staining (red) (**E**), or immunohistochemical staining using anti-BrdU (brown) antibody (**G**). (**G**) Filled and blank arrowheads indicate BrdU-positive tubular and interstitial cells, respectively. Collagen-deposited area (%) (**F**) and BrdU-positive (BrdU^+^) tubular (**H**) and interstitial (I) cell numbers were measured as described in the Materials & Methods. Results are expressed as mean ± SEM (n = 5). *p < 0.05 vs. respective sham-vehicle.

To investigate whether tubulin deacetylation affects tubule cell regeneration and progression of fibrosis, colchicine, a well-known inhibitor of tubulin acetylation by depolymerizing microtubule^15^,^22^, was administrated to mice every other day during the recovery phase after I/R insult. Post-I/R reduction of acetylated α-tubulin expression was greater in colchicine-treated mice than in vehicle-treated mice (Fig. 9A,B). Post-I/R increases in p21 and α-SMA expression were greater in colchicine-treated mouse kidneys than in vehicle-treated mouse kidneys (Fig. 9A,C,D). Collagen deposition was greater in colchicine-treated mouse kidneys than in vehicle-treated mouse kidneys (Fig. 9E,F). Treatment of colchicine significantly reduced the post-I/R increases in BrdU-positive tubule cells, whereas it enhanced the post-I/R increase in BrdU-positive interstitial cells (Fig. 9G–I). Taken together, these data indicate that inhibition of microtubule dynamics induced by increased tubulin deacetylation delay recovery of damaged kidney, resulting in great fibrosis.

## Discussion

In the present study, we report for the first time that I/R injury causes α-tubulin deacetylation in microtubules, and that inhibition of microtubule dynamics induced by the changes of tubulin acetylation and deacetylation during the recovery phase retards tubule cell regeneration. These findings suggest that the regulation of microtubule dynamics by post-translational modifications could be considered as a treatment modality in AKI and CKD.

Acetylation and deacetylation of α-tubulin regulates the microtubule dynamics by increasing and decreasing microtubule stability, respectively^15^. Microtubule dynamics are indispensable for cell differentiation and proliferation^17^,^23^,^24^. When cells enter mitosis, the interphase cytoskeletal microtubules are disassembled, and a bipolar spindle is created. Spindle microtubules attach to chromosomes at the kinetochore and contribute to chromosome alignment, and subsequent segregation during anaphase^25^. In cancer cells, it has been reported that inhibition of microtubule dynamics by taxol disrupts the mitotic spindle assembly and alignment of chromosomes during mitosis, leading to cell apoptosis^26^. In the present study, we found that normal kidney cell cytoplasm exhibits typical microtubule networks arranged in a basal-to-apical orientation, whereas kidney cells recovering from I/R injury present an unstable and scattered microtubule network. These results indicate that inhibition of microtubule dynamics by changes of tubulin acetylation and deacetylation retards renal recovery following I/R injury. Indeed, in the present study, taxol and cholchicine suppressed tubule cell proliferation and accelerated fibrosis.

Unlike our results, Zhang *et al.* reported that cell cycle arrest by taxol suppressed progression of renal fibrosis in a rat UUO model^4^. This apparent discrepancy between our result and those of Zhang *et al.* may be due to the timing of taxol injections, and differences in the experimental animal model. Zhang *et al.* treated the animals with taxol from initial injury to fibrosis, whereas we treated with taxol during the recovery phase, and only after the peak of injury^4^. Therefore, taxol pretreatment prior to injury may enhance microtubule stability, resulting in a greater degree of renal cell injury protection and reduced fibrosis. In addition, we speculated that taxol treatment in our experiments may minimally affect injuring cells, whereas it may maximally affect recovering, dividing, and differentiating cells, and that taxol could delay repair of damaged tubule cells through microtubule stabilization, resulting in cell cycle arrest and fibrosis. Moreover, as shown in our study, post-treatment with taxol may inhibit microtubule dynamics, resulting in impaired tubule cell regeneration and subsequent fibrosis.

Interestingly, taxol inhibits tubule cell proliferation in the post-I/R kidney, whereas it accelerated interstitial cell proliferation. This distinction in the effects of taxol on cell proliferation between the groups may be due to cell type. To test this, we compared the *in vitro* effect of TTx on murine tubular epithelial cells and murine monocytes/macrophages. Based upon previous studies demonstrating that bone marrow-derived inflammatory cells are a major interstitial cell component in post-I/R and post-UUO fibrotic kidneys^20^,^21^, we selected monocytes/macrophages as a representative interstitial cell type. In the present study, low concentration TTx induced tubulin acetylation in both tubular epithelial cells and monocytes/macrophages, whereas high concentration TTx induced tubulin acetylation only in tubule cells, but not in monocytes/macrophages. We found that acetylated-α-tubulin expression *in vivo* after I/R insult was higher in taxol-treated mouse tubular epithelial cells than that in non-taxol-treated tubular epithelial cells, but not in interstitial cells. Therefore, the opposing effects of TTx on the post-I/R proliferation of tubule cells and interstitial cells may be associated with cell-type. It is possible that because Raw cells are relatively less responsive to taxol, the increased population of proliferating interstitial cells after TTx may be due to increased inflammatory cell infiltration and proliferation in response to necrotic debris from damaged tubule cells^20^,^21^. Supporting this, PCNA in 16 day post-I/R fibrotic kidney was mainly observed in the interstitial cells.

In the present study, we found that I/R injury decreased the expression of αTAT1 with recovery not occurring until nine days after the ischemic event. In contrast, the expression of HDAC6 was only minimally altered after I/R injury. The HDAC6 levels were slightly reduced three days after ischemia onset, and recovery was observed on day nine. We found that TTx affects *in vitro* α-TAT1 and HDAC6 expression in the tubule cells, although it did not induce statistically significant changes in the monocytes/macrophages. These results indicate that I/R injury affects tubulin deacetylation mainly via a decrease in α-TAT1 expression, suggesting that the regulation of microtubule acetylation by those enzymes is associated with fibrosis. Andrea-Aguilar *et al.* recently reported that αTAT1 deficiency results in a loss of contact-dependent growth inhibition, leading to cell overgrowth and the formation of multiple cell layers in culture dishes. The authors suggest that microtubule deacetylation accelerates abnormal cell growth^27^. Recently, Choi *et al.* showed that HDAC6 inhibition by tubastatin A prevented fibrosis induced by angiotensin II infusion^28^, and Jordan *et al.* reported that taxol-induced suppression of microtubule dynamics inhibits cell proliferation^29^.

Several studies have demonstrated that cell cycle arrest of injured tubular epithelial cells promotes the progression of renal fibrosis^5^, and taxol arrests the cell cycle at the G0/G1 and G2/M phases leading to inhibition of cell proliferation by polymerization, and subsequent stabilization of microtubules^30^,^31^,^32^,^33^. Chk2 stabilizes p53, leading to cell cycle arrest in the G1 phase. As a cyclin-dependent kinase (CDK) inhibitor, p21 inhibits the CDK2 and CDK1 complexes, and induces cell cycle arrest^34^. In the present study, we found that TTx induced increases in p-chk2 and p21 expression in both Sham and I/R-injured kidneys. In addition, TTx induced *in vitro* cell cycle arrest in tubule cells, but not in monocytes/macrophages. Therefore, delayed tubular epithelial cell regeneration and worsened kidney fibrosis may be caused, in part, by cell cycle arrest in tubular cells. Furthermore, tubulin deacetylation by colchicine, a microtubule depolymerizing agent, had shown similar effect with taxol on cell cycle, proliferation and progression of renal fibrosis. This suggests that disruption of balance between microtubule polymerization and depolymerization may inhibit microtubule dynamics, leading to tubular cell cycle arrest and delayed tubule cell regeneration. Supporting this, taxol treatment suppressed cell proliferation after H_2_O_2_ injury in the cultured tubular epithelial cells.

In summary, our results have demonstrated that inhibition of microtubule dynamics during the recovery phase after I/R injury results in delayed functional and morphological renal recovery. This leads to accelerated fibrosis, suggesting that the regulation of microtubule dynamics could represent a preventive and therapeutic strategy for AKI and CKD.

## Materials and Methods

### Animal preparation

Eight-week-old C57BL/6 male mice (Koatech, Gyounggido, Korea) were used in these experiments. All studies were conducted in accordance with the guidelines provided by the Animal Care and Use Committee of Kyungpook National University and were conducted in accordance with the Guide for the Care and Use of Laboratory Animals, published by the US National Institutes of Health (NIH Publication No. 85–23, revised 2011). For the induction of ischemia, the kidney was exposed through a flank incision under anesthetization with pentobarbital sodium (50 mg/kg BW), and the renal pedicle was clamped completely for 30 min using a microaneurysm clamp. The same procedure, exclusive of renal pedicle clamping, was performed in the sham operation (Sham). Mice received either intraperitoneal (IP) taxol treatment (TTx) (0.3 mg/kg BW, Enzo Life Science, Farmingdale, NY, USA) or colchicine (0.1 mg/kg BW, Enzo Life Science, Farmingdale, NY, USA) or saline (vehicle) beginning on day one following ischemia induction, and continuing every other day until sacrifice. A minimum of six mice were included in each experimental group. For biochemical and histological analyses, the kidneys were either frozen in liquid nitrogen or perfusion-fixed with periodate-lysine-paraformaldehyde (PLP) (4% paraformaldehyde, 75 mM l-lysine, 10 mM sodium periodate; Sigma-Aldrich Corporation, St. Louis, MO, USA) immediately following retrieval.

### Cell experiments

Murine proximal tubular epithelial cells (mProx24) derived from C57BL/6J adult mouse kidney as described previously^35^ were gifted by Dr. Kwon HM (UNIST, Ulsan, Korea). RAW 264.7 cells, a murine macrophage/monocyte were purchased from ATCC (ATCC® TIB-71™). mProx24 and RAW264.7 cells were cultured in Dulbecco’s modified Eagle’s medium (DMEM) with 15% fetal bovine serum (FBS) (Mediatech Inc., Manassas, VA, USA) with 100 u/ml streptomycin/penicillin (S/P) (WelGENE Inc., Daegu, Korea). mProx24 and RAW264.7 cells were seeded on 0.1% gelatin-coated culture dishes and normal culture dishes (Falcon, Oxnard, CA, USA), respectively. To determine mitotic cells, cells were fixed (4% paraformaldehyde) and then subjected to immunofluorescence**-**staining to evaluate mitotic spindle using anti-α-tubulin antibody. DAPI stain was used to visualize the nucleus. Proliferation of cells was evaluated mitotic spindle-stained by α-tubulin antibody and dividing nucleus by DAPI. Number of cells and mitotic cells were counted within 10 fields per slide.

### Renal functional parameters

Blood samples (70 μL) were obtained from the retro-orbital venous plexus. Plasma creatinine (PCr) and blood urea nitrogen (BUN) concentrations were measured with a Vitros 250 (Johnson & Johnson, New Brunswick, NJ, USA).

### BrdU-incorporation assay

To detect *in vivo* cell proliferation, we performed a 5-bromo-2′-deoxyuridine (BrdU) incorporation assay. As a thymidine analog, BrdU is incorporated into newly synthesized DNA during the S phase of cell replication^36^. BrdU (50 mg/kg body weight; Sigma-Aldrich Corporation, St. Louis, MO, USA) was administered IP to mice beginning on day one following either ischemia induction or Sham, and continued every other day until sacrifice. Following retrieval, kidney sections were subjected to immunohistochemical or immunofluorescence staining using anti-BrdU antibody. Photomicrographs (400X magnification) of the cortical and outer medullary regions were captured using a Leica microscope (DM2500, Wetzlar, Germany).

### Western blot analysis

Western blot analysis was performed as previously described^37^ utilizing the following antibodies: anti-α-smooth muscle actin (anti-α-SMA) (1:20,000 dilution, Sigma-Aldrich Corporation, St Louis, MO, USA), anti-vimentin (1:1,000 dilution, Sigma-Aldrich Corporation, St Louis, MO, USA), anti-phosphorylated-chk2 (anti-p-chk2) (1:1,000 dilution, Cell Signaling Technology, Inc., Danvers, MA, USA), anti-p21 (1:1,000 dilution; Santa Cruz Biotechnology, Inc., Santa Cruz, CA, USA), anti-proliferating cell nuclear antigen (anti-PCNA) (1:20,000 dilution, DAKO, Carpinteria, CA, USA), anti-β-actin (1:20,000 dilution, Sigma-Aldrich Corporation, St Louis, MO, USA), anti-acetylated α-tubulin (anti-ac-α-tubulin) (1:10,000 dilution, Sigma-Aldrich Corporation, St Louis, MO, USA), anti-α-tubulin (1:10,000 dilution, Santa Cruz Biotechnology, Inc., Santa Cruz, CA, USA), anti-α-tubulin acetyltransferase 1 (anti-α-TAT1) (1:1,000 dilution, Santa Cruz Biotechnology, Inc., Santa Cruz, CA, USA), anti-histone deacetylase 6 (anti-HDAC6) (1:2,000 dilution, Santa Cruz Biotechnology, Inc., Santa Cruz, CA, USA), and anti-glyceraldehyde-3-phosphate dehydrogenase (anti-GAPDH) (1:20,000 dilution; Novus Biologicals LLC, Littleton, CO, USA).

### Histology

Paraffin-embedded kidney tissue sections were stained with Periodic Acid-Schiff (PAS) stain according to a standard protocol. The degree of morphological tubular damage was scored within 10 fields per kidney section^38^.

As described previously^18^, immunohistochemical staining was performed using the following Abs: anti-BrdU (1:100 dilution; Abcam PLC, Cambridge, UK), anti-type III collagen-UNLB (anti-collagen-III) (1:100 dilution; Southern Biotech, Birmingham, AL, USA), anti-F4/80 (1:100 dilution; Serotec, Oxford, UK), anti-PCNA (1:300 dilution, DAKO, Carpinteria, CA, USA), and anti-ac-α-tubulin (1:200 dilution, Sigma-Aldrich Corporation, St Louis, MO, USA). Sections were observed under a Leica microscope (DM2500, Wetzlar, Germany). Immunofluorescence staining was performed using anti-ac-α-tubulin (1:100, Santa Cruz Biotechnology, Inc., Santa Cruz, CA, USA) and anti-α-tubulin (1:100, Santa Cruz Biotechnology, Inc., Santa Cruz, CA, USA) antibodies as described previously^18^. Sections were viewed with a Leica microscope (DM2500, Wetzlar, Germany). Aggregation of ac-α-tubulin expression in cells was measured within 200 tubule cells per kidney using an image analysis program (i-solution, IMT, Korea).

### Picro-sirius red staining

Paraffin-embedded kidney tissue sections were stained with picro-sirius red (PSR) according to a standard protocol. Dewaxed kidney tissue sections were exposed to PSR stain for 1 h, and then washed twice with acidified water (0.5% glacial acetic acid). Sections were then serially dehydrated in different alcohol concentrations. Photomicrographs (400X magnification) were obtained randomly from the outer medullary region using a Leica microscope (DM2500, Wetzlar, Germany). Regions of collagen deposition in the PSR stained kidney sections were measured using an image analysis program (i-solution, IMT, Korea).

### Statistical analysis

All results are expressed as mean ± SEM. Statistical differences between groups were determined using the Students *t*-test. A p value less than 0.05 was considered significant.

All experiments were performed in accordance with relevant guidelines and regulation. All experimental protocols described in this paper were approved by Kyungpook National University. All animal studies were carried out in the restricted facilities provided by the Kyungpook National University, by authorized personnel following the prescribed rules.

## Additional Information

**How to cite this article**: Han, S. J. *et al.* Inhibition of microtubule dynamics impedes repair of kidney ischemia/reperfusion injury and increases fibrosis. *Sci. Rep.* **6**, 27775; doi: 10.1038/srep27775 (2016).
